# Imaging in localized intrahepatic bile duct dilatation: a pictorial review of diagnostic and differential approaches

**DOI:** 10.1186/s13244-026-02419-w

**Published:** 2026-10-09

**Authors:** Yongyi Cen, Xiaoqi Zhou, Mimi Tang, Yanjin Qin, Chenyu Song, Jifei Wang, Yanji Luo, Zhi Dong, Hongyan Dai, Zhibing Ruan, Zhenpeng Peng, Shi-Ting Feng

**Affiliations:** 1 Department of Radiology, The First Affiliated Hospital, Sun Yat-Sen University, Guangzhou, China; 2 Department of Radiology, Affiliated Hospital of Youjiang Medical University for Nationalities, Baise, China; 3 Department of Radiology, The Affiliated Hospital of Yunnan University, Kunming, China; 4 Department of Radiology, The Affiliated Hospital of Guizhou Medical University, Guiyang, China

**Keywords:** Localized intrahepatic bile duct dilatation, Imaging, Computed tomography, Magnetic resonance imaging, Differentiation

## Abstract

**Abstract:**

Localized intrahepatic bile duct dilatation (LIBD) is a prevalent radiological finding characterized by a variety of etiologies, including congenital anomalies, inflammatory conditions, hepatolithiasis, and neoplastic lesions. Accurate differential diagnosis of diseases associated with LIBD is crucial for formulating appropriate treatment strategies and improving patient prognosis. This review provides a comprehensive overview of the definitions, etiologies, clinical symptoms, and key imaging features of common diseases associated with LIBD. Furthermore, it proposes a structured approach for differential diagnosis. By integrating imaging findings with clinical and laboratory data, this approach aims to improve diagnostic accuracy and assist radiologists in making optimal clinical decisions.

**Key Points:**

***Question*** LIBD has diverse etiologies and overlapping imaging patterns, but a practical, standardized radiological framework for differential diagnosis remains lacking.

***Findings*** This pictorial review summarizes characteristic CT/MRI findings of common LIBD-associated diseases and establishes a stepwise diagnostic approach integrating obstruction points, morphology, and clinical information.

***Critical Relevance Statement*** By combining specific imaging patterns with clinical context, this review provides a practical differential diagnostic framework that may improve diagnostic confidence, guide further evaluation, and facilitate earlier detection of malignant transformation in LIBD.

**Graphical Abstract:**

**Figure:**
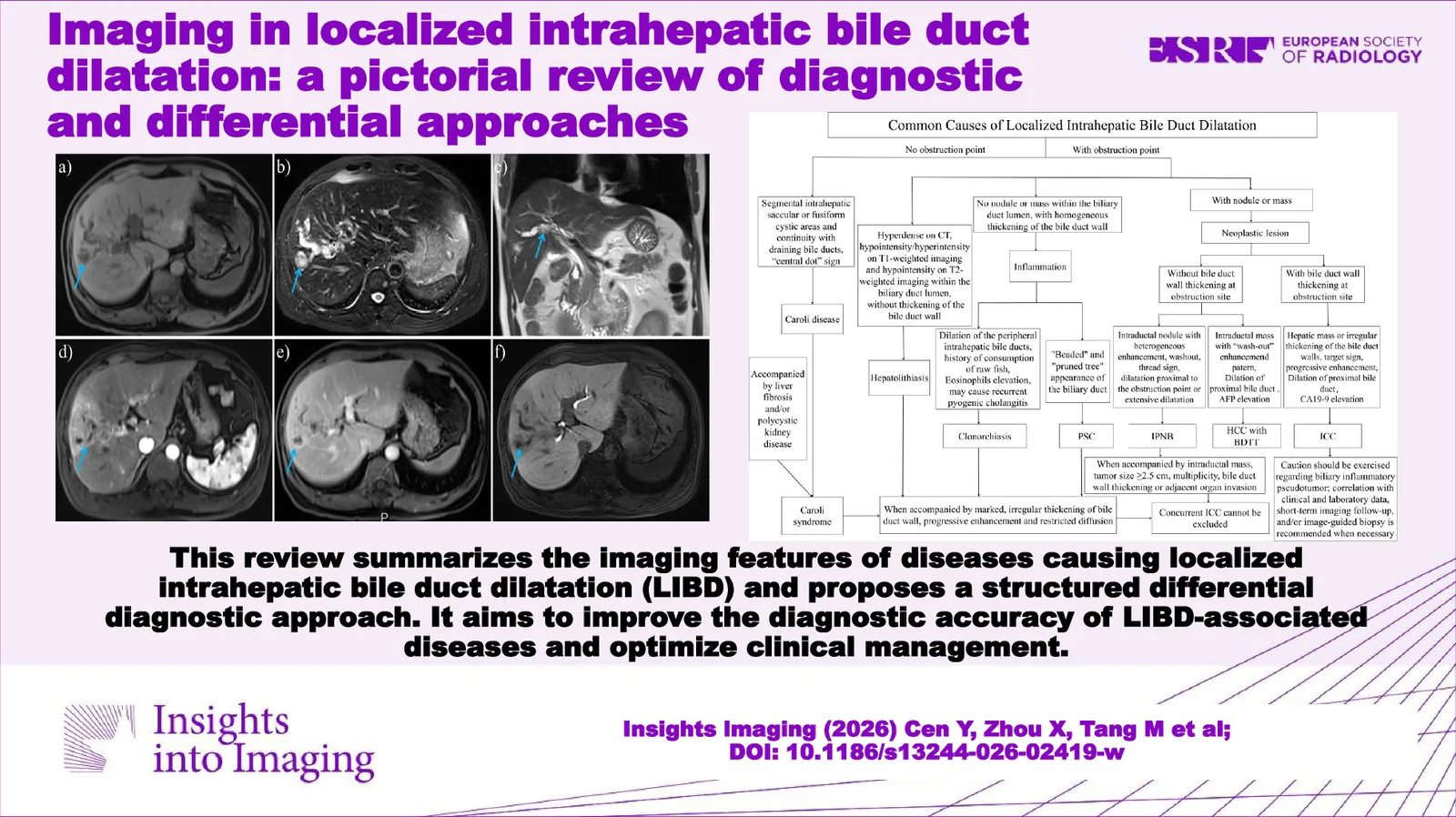

## Introduction

Localized intrahepatic bile duct dilatation (LIBD) is characterized by the saccular or fusiform enlargement of focal intrahepatic biliary segments. It is a common yet challenging radiological finding in clinical practice. The underlying etiologies of LIBD are highly heterogeneous and include congenital anomalies (e.g., Caroli disease (CD)/syndrome) [1, 2], inflammatory conditions (e.g., clonorchiasis, primary sclerosing cholangitis (PSC)) [3, 4], hepatolithiasis [5], and neoplastic lesions (e.g., intrahepatic cholangiocarcinoma (ICC), intraductal papillary neoplasm of the bile duct (IPNB)) [6–8]. Considering the different therapeutic strategies and prognostic implications associated with these disorders, precise etiological identification is essential for guiding optimal clinical management.

Despite advancements in CT and MRI, which have markedly improved the visualization of biliary tract abnormalities, a standardized, evidence-based framework for differentiating LIBD-associated diseases is still lacking in current clinical practice. This gap in knowledge often leads to diagnostic uncertainty, potentially delaying appropriate treatment and compromising patient outcomes.

This review summarizes the clinical characteristics, etiology, and key CT/MRI imaging features of common LIBD-associated diseases, and further proposes a structured differential diagnostic approach. The objective is to provide radiologists with a comprehensive and practical guide to improve diagnostic accuracy and facilitate informed clinical decision-making.

## Normal anatomy and dilation of the intrahepatic bile ducts

**Fig. 1:**
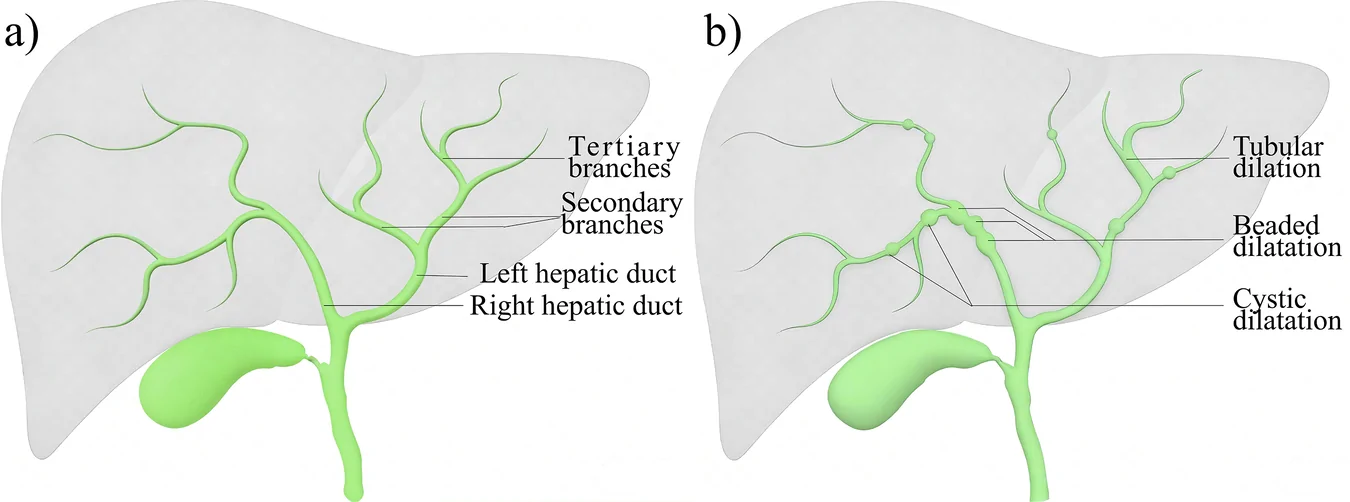
Diagram of Normal Intrahepatic Biliary Anatomy and Intrahepatic Biliary Dilation. **a** Depicts the morphology and course of normal intrahepatic bile ducts. **b** Illustrates pathological dilation, manifesting as three classic patterns: cystic, tubular, and beaded dilatation

The intrahepatic bile ducts primarily collect and transport bile produced by hepatocytes, and accurate identification of their anatomy is essential for assessing bile duct dilation and differentiating potential causes. This ductal system originates from capillary bile ducts within liver lobules, which merge through interlobular bile ducts to form a hierarchical branching structure that aligns with the liver’s functional zones. The primary branches, or first-order branches, consist of the left and right hepatic ducts, accompanied by the hepatic artery and portal vein within Glisson’s capsule. Secondary branches correspond to the liver lobar bile ducts, while tertiary branches correspond to the bile ducts of the Couinaud segments (S1–S8) (Fig. 1). On imaging, the rapid identification of bile duct branching abnormalities provides an anatomical reference essential for etiological analysis.

In general, for individuals under 60 years of age, the diagnostic criterion for intrahepatic bile duct dilatation is defined as a distal bile duct diameter greater than 2 mm or a diameter exceeding 40% of the diameter of the adjacent portal vein [9]. Pathological bile duct dilatation can manifest in various forms, such as cystic, tubular, or beaded appearances (Fig. 1). These morphological variations are often associated with specific etiologies, necessitating further clinical differentiation. With advancing age, the elasticity of bile duct walls decreases, which may lead to a physiological widening of the bile duct diameter. However, this widening occurs slowly, at a rate of 0.3-0.7 mm per decade [10], and is typically not associated with bile duct wall thickening or signs of cholestasis, making it distinguishable from pathological dilatation.

## Definition, etiology, clinical symptoms, and imaging findings of common LIBD-associated diseases

### CD and Caroli syndrome (CS)

#### Definition and etiology

CD is a rare congenital disorder characterized by cystic dilation of the intrahepatic bile ducts [11]. Isolated CD is uncommon, as most patients present with concomitant congenital hepatic fibrosis, which increases intrahepatic vascular resistance and results in portal hypertension, a condition known as CS [11, 12].

Studies have demonstrated that CD is associated with ductal plate malformations during embryonic development. These malformations hinder the proper formation of large intrahepatic bile ducts around the 12th week of gestation, contributing to the pathogenesis of CD [13, 14]. In CS, ductal plate malformations may persist into later developmental stages, affecting the development of peripheral and small bile ducts. Unlike CD, CS is inherited in an autosomal recessive pattern and often coexists with autosomal recessive polycystic kidney disease (ARPKD). Both CS and ARPKD are associated with mutations in the PKHD1 gene, located on chromosome 6 (6p12.3–p12.2) [15].

#### Clinical symptoms

Most patients with CD present with symptoms before the age of 30, with diagnosis often made after symptom onset. Clinical manifestations include fever, abdominal pain, and jaundice, typically resulting from complications such as cholangitis and hepatolithiasis. In some cases, cholestasis-induced hyperbilirubinemia can result in pruritus. Patients with CS commonly present with symptoms of portal hypertension and renal insufficiency, including hepatosplenomegaly, ascites, esophageal variceal bleeding, and renal failure. A significant concern in both CD and CS is the increased risk of cholangiocarcinoma, with an estimated prevalence of 7% among affected individuals [16]. Accordingly, the European Association for the Study of the Liver recommends annual surveillance with magnetic resonance cholangiopancreatography (MRCP) following diagnosis [1].

#### Imaging findings

**Fig. 2:**
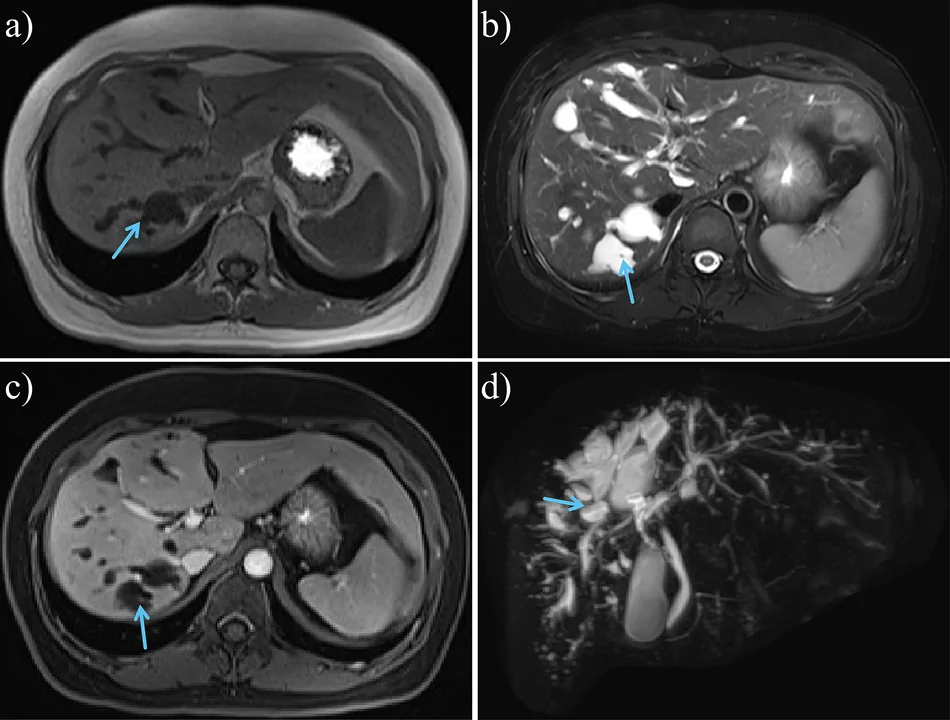
Imaging findings in a 37-year-old female with CD. T1-weighted imaging (**a**) and MRCP (**d**) demonstrate cystic dilation of the intrahepatic bile ducts, which communicate with the biliary tree (arrow). T2-weighted imaging (**b**) and gadoxetic acid disodium-enhanced portal venous phase imaging (**c**) reveal the “central dot” sign, a characteristic finding frequently identified in CD (arrow)

CD and CS are characterized by the cystic dilatation of intrahepatic bile ducts that communicate with the normal biliary tree (Fig. 2). This feature can be clearly visualized using MRCP or hepatobiliary-specific contrast agents such as gadoxetic acid and gadobenate dimeglumine [1, 17]. An additional diagnostic indicator is the “central dot” sign, observable on contrast-enhanced CT or MRI. It presents as a small, enhancing portal vein or hepatic artery branch located within the dilated intrahepatic bile duct lumen [17]. This sign is typically not observed in other conditions without biliary obstruction, such as biliary necrosis and portal biliopathy (Fig. 2). However, it is important to note that this finding is not pathognomonic [1, 17].

A critical and diagnostically valuable feature of CD/CS on gadoxetic acid- or gadobenate dimeglumine-enhanced MRI is the excretion of contrast agent into the saccular dilatations, which confirms continuity between normal bile ducts and cystic lesions. However, the low hepatobiliary excretion fraction and prolonged excretion duration of gadobenate dimeglumine make this feature challenging to detect. For gadoxetic acid, the delay time for visualizing hepatobiliary excretion is typically 20 min or more after contrast administration, allowing sufficient time for the contrast agent to be taken up by hepatocytes and excreted into the biliary system, including the dilated ducts [18]. This finding helps distinguish CD/CS from other cystic liver lesions that do not communicate with the biliary tree and thus do not show gadoxetic acid filling. Notably, intrahepatic biliary abnormalities caused by CD and CS do not exhibit features such as intraductal filling defects or extrinsic biliary compression.

**Fig. 3:**
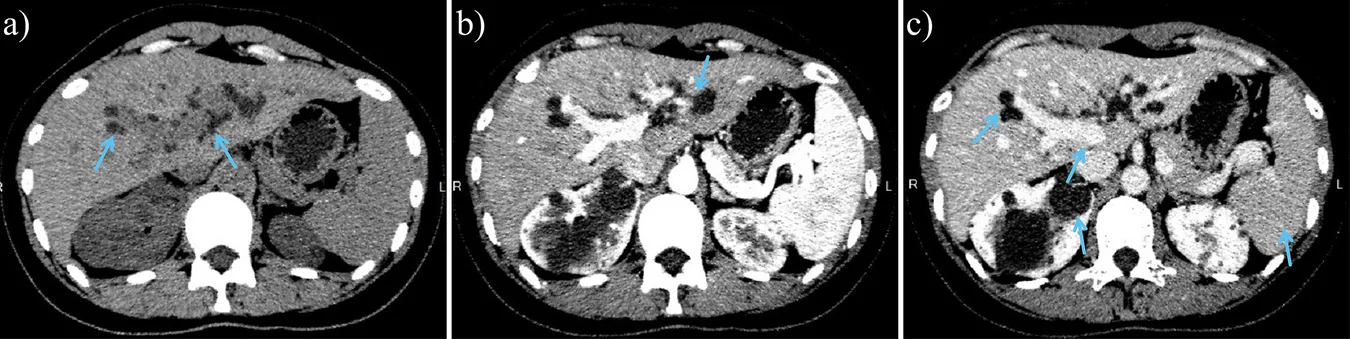
Imaging findings in a 29-year-old female with CS. Unenhanced computed tomography (CT) (**a**), arterial phase CT (**b**), and portal venous phase CT (**c**) show localized cystic dilation of the intrahepatic bile ducts, which communicate with the bile duct tree (arrow). Additional findings include polycystic kidneys, portal vein enlargement, and splenomegaly (**c**, arrows)

In CS, additional imaging findings may include hepatic fibrosis, portal hypertension, splenomegaly, esophageal varices, and features of polycystic kidney disease [1] (Fig. 3). CT is useful for detecting gross anatomical abnormalities such as splenomegaly and polycystic kidneys, while MRI is more sensitive for evaluating hepatic fibrosis and portal hypertension-related changes. While ultrasound and CT are frequently used as initial screening modalities for CD and CS in clinical practice, MRI with MRCP remains the preferred imaging modality for definitive lesion characterization and confirmatory diagnosis.

### Hepatolithiasis

#### Definition and etiology

Hepatolithiasis refers to the presence of biliary stones within the intrahepatic bile ducts, located above the confluence of the left and right hepatic ducts [19, 20]. This disease is endemic to East and Southeast Asia, where prevalence rates have been reported to be as high as 30% [19]. The etiology of hepatolithiasis is multifactorial, involving biliary anatomical abnormalities, bile infection, cholestasis, and hyperplasia of the biliary epithelium. These mechanisms contribute to an increased risk of cholangiocarcinoma in affected patients [21–23].

#### Clinical symptoms

The clinical manifestations of hepatolithiasis vary depending on the presence of biliary obstruction and infection, as well as progression to secondary biliary cirrhosis and hepatic decompensation. Some patients may remain asymptomatic or experience only mild abdominal discomfort, whereas those with biliary infection often develop more severe symptoms. These include abdominal pain, fever, and jaundice, collectively known as Charcot’s triad. In severe cases, the condition may progress to Reynold’s pentad, characterized by abdominal pain, fever, jaundice, confusion, and hypotension. In advanced stages, chronic biliary obstruction and recurrent infections lead to periductal hepatic fibrosis, subsequently resulting in secondary biliary cirrhosis and ultimately progressing to hepatic decompensation, which manifests as persistent jaundice and refractory ascites. The primary treatment of hepatolithiasis involves interventional approaches aimed at alleviating cholestasis and removing impacted stones. For patients with advanced biliary strictures and concurrent hepatic parenchymal atrophy, liver resection remains a cornerstone of therapy.

#### Imaging findings

In general, hepatolithiasis obstructs the bile duct lumen and impairs bile drainage, resulting in elevated intraductal pressure and upstream bile duct dilatation. Persistent hepatolithiasis stimulates the ductal wall and triggers inflammatory reactions, leading to mural edema, fibrosis, or thickening, which further promotes dilatation of the biliary system.

**Fig. 4:**
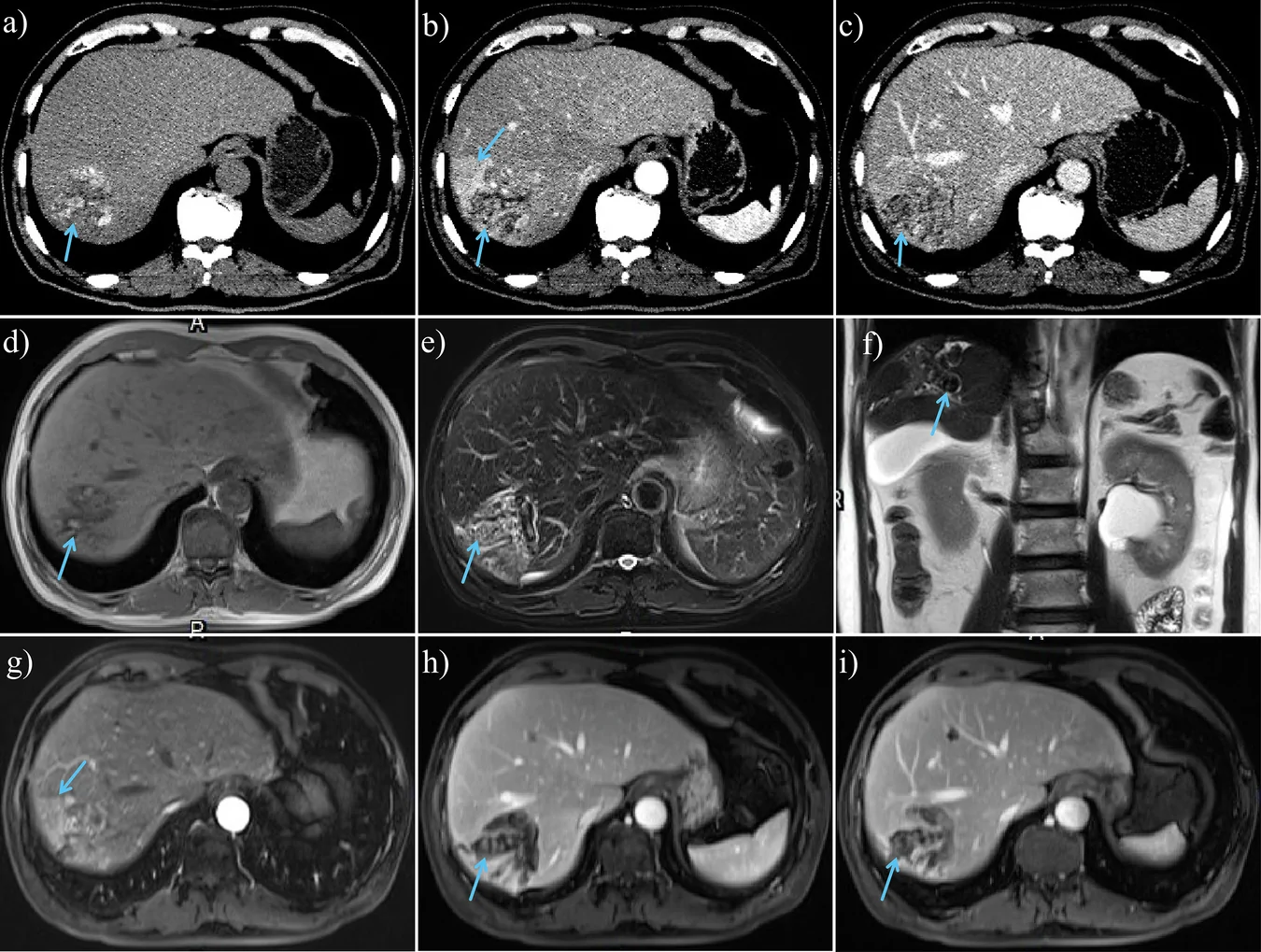
Imaging findings in a 67-year-old man with multiple hepatolithiasis. Unenhanced CT (**a**), arterial phase CT (**b**), and portal venous phase CT (**c**) show hepatolithiasis as areas of hyperattenuation or isoattenuation (arrow). On T1-weighted imaging (**d**), the hepatolithiasis exhibits variable signal intensities, whereas axial (**e**) and coronal (**f**) T2-weighted imaging show the hepatolithiasis as hypointense (arrow). Arterial phase magnetic resonance imaging (MRI) (**g**), portal venous phase MRI (**h**), and delayed phase MRI (**i**) with gadoxetic acid disodium reveal no enhancement of the hepatolithiasis (arrows). Additionally, the arterial phase images from both CT (**b**) and MRI (**g**) display transient enhancement of the adjacent hepatic parenchyma (arrows)

Some hepatolithiasis lesions are isoattenuating to bile, making them difficult to detect on conventional CT images. However, stones with substantial calcified components show hyperattenuation and may appear cast-like, conforming to the biliary tract (Fig. 4). Dual-energy CT enhances the visualization of stones by generating material-specific images, which improve the detection of both calcified and non-calcified stones compared to conventional CT, thereby increasing the detection rate of biliary calculi [24].

On T2-weighted imaging and MRCP, biliary stones appear hypointense, while showing variable signal intensity on T1-weighted imaging, with signal variability dependent on their composition (Fig. 4). Cholesterol stones are hypointense, whereas pigmented stones are hyperintense on T1-weighted images [17]. MRCP offers significant advantages for the detection of hepatolithiasis [5], enabling precise evaluation of bile duct obstruction, the degree of dilatation caused by stones, and the relationship between stones and bile duct strictures. Notably, in some patients, transient perihepatic parenchymal enhancement may be observed in the arterial phase of CT or MRI (Fig. 4). This finding is likely attributable to congestion and hyperperfusion driven by the inflammatory response.

In summary, CT is superior to MRI for detecting calcified hepatolithiasis, while MRI/MRCP is more sensitive for detecting non-calcified stones and evaluating the anatomy of the biliary tree. Ultrasound serves as the first-line screening modality for hepatolithiasis, whereas MRCP is the preferred imaging technique for preoperative diagnosis and comprehensive evaluation of this condition.

### Clonorchiasis

#### Definition and etiology

Clonorchiasis is a parasitic infection caused by the consumption of raw or undercooked freshwater fish or shrimp contaminated with infective larvae. After ingestion, the larvae excyst in the duodenum, ascend into the biliary tree, and mature into adult worms, establishing themselves in the biliary tract, gallbladder, or liver [25, 26]. This infection is highly prevalent in several regions, including China, South Korea, northern Vietnam, and the far eastern region of Russia, with an estimated 15 million people affected worldwide [25].

#### Clinical symptoms

Clonorchiasis is often asymptomatic or presents with mild gastrointestinal disturbances, such as indigestion, nausea, diarrhea, and abdominal discomfort. The lack of characteristic manifestations often delays clinical recognition and treatment, increasing the risk of persistent infection. Long-term infection with Clonorchis sinensis can cause progressive damage to the biliary tract, potentially leading to bile duct obstruction, recurrent pyogenic cholangitis, cholelithiasis, and, in severe cases, cholangiocarcinoma [3]. Most affected individuals also exhibit peripheral eosinophilia. Currently, no vaccine for clonorchiasis has been developed, and praziquantel remains the cornerstone of morbidity control. Complementary measures, such as environmental modifications to prevent fecal contamination and community education to discourage the consumption of raw freshwater fish, are essential for enhancing both the efficacy and sustainability of control programs [25].

#### Imaging findings

**Fig. 5:**
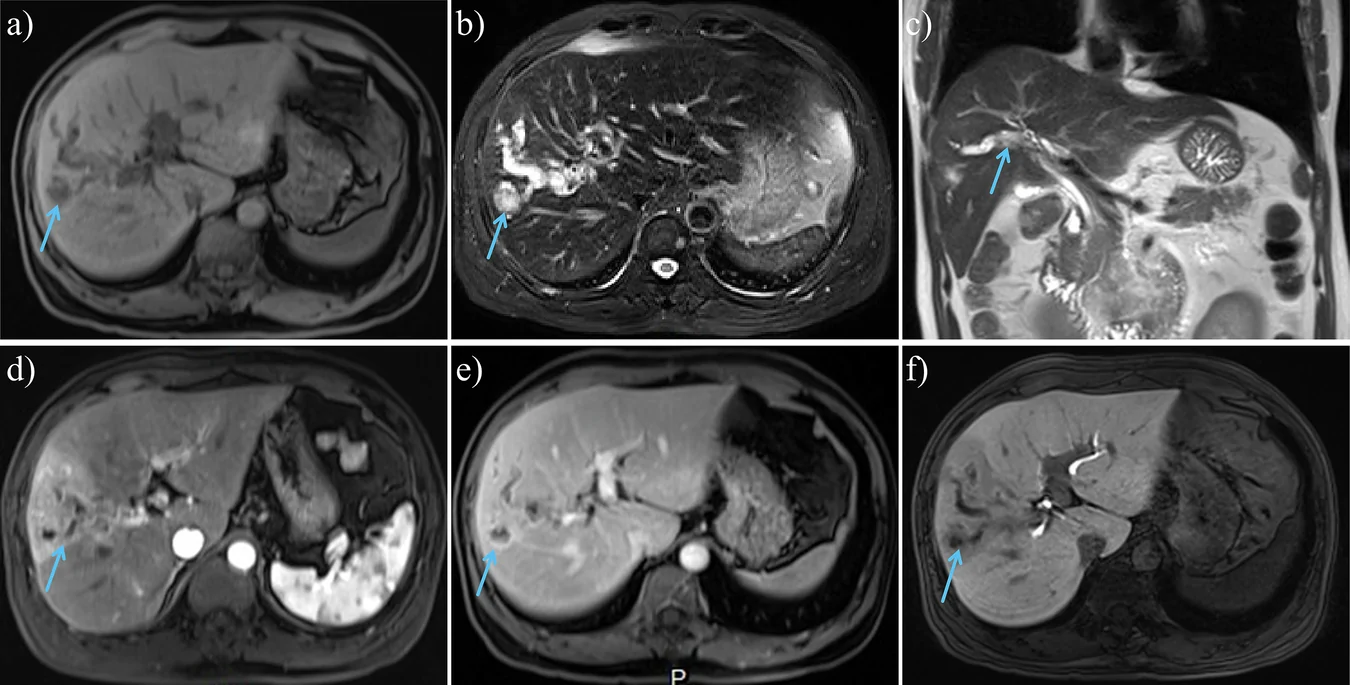
Imaging findings in a 56-year-old man with clonorchiasis. T1-weighted imaging (**a**) demonstrates LIBD in the right hepatic lobe. Axial (**b**) and coronal (**c**) T2-weighted images both reveal filling defects within the dilated bile ducts. Arterial-phase (**d**), portal venous-phase (**e**), and hepatobiliary-phase MRI (**f**) all show no enhancement of these intraductal filling defects, whereas portal venous-phase MRI (**e**) most prominently demonstrates homogeneous thickening and enhancement of the bile duct wall (arrows)

Owing to the aggregation of numerous parasites and the inflammatory response of the bile duct wall induced by parasite secretory metabolites, clonorchiasis typically manifests as localized or diffuse dilation of the peripheral intrahepatic bile ducts on CT, with or without mild ectasia of the extrahepatic bile ducts. MRI provides additional detail, revealing homogeneous wall thickening with enhancement and small filling defects in the bile ducts [27] (Fig. 5). T2-weighted imaging and MRCP may show filling defects within the dilated ducts, corresponding to the presence of worms [28]. These imaging features can overlap with those of periductal-infiltrating cholangiocarcinoma and PSC. However, unlike periductal-infiltrating cholangiocarcinoma, clonorchiasis does not cause irregular bile duct wall thickening, aggressive strictures, or soft tissue masses. Compared to PSC, clonorchiasis tends to involve more peripheral intrahepatic bile ducts and lacks the characteristic “beaded” or “pruned tree” appearance of bile ducts. Notably, in clonorchiasis cases, follow-up imaging after treatment with praziquantel often shows reduced dilation of bile ducts and decreased bile duct wall thickness, providing both diagnostic confirmation and evidence of disease response.

MRI is generally preferred over CT for the diagnosis of clonorchiasis due to its superior soft tissue contrast, ability to detect small filling defects, and mild wall thickening. MRCP is particularly useful for evaluating the extent of biliary dilation and identifying filling defects.

### PSC

#### Definition and etiology

PSC is a chronic cholangiopathy characterized by periductular fibrosis affecting both intrahepatic and extrahepatic bile ducts. This pathological process drives the development of biliary strictures, recurrent bacterial cholangitis, and progressive liver decompensation, often necessitating liver transplantation [29]. PSC is a rare disease with a predilection for young males and is strongly associated with inflammatory bowel disease (IBD), which is observed in the majority of patients [4, 29, 30]. While the etiology of PSC remains unclear, it is believed to be closely linked to autoimmune conditions [31]. Moreover, the disease is associated with an elevated risk of various malignancies, most notably cholangiocellular carcinoma, although the mechanisms underlying this risk are not well understood [29].

#### Clinical symptoms

Early-stage PSC is typically asymptomatic and is often detected incidentally during routine examinations or IBD screening. Approximately half of the patients eventually develop symptoms such as right upper quadrant pain, jaundice, pruritus, fatigue, fever, or weight loss [32, 33]. Notably, abdominal pain and jaundice often present as fluctuating and recurrent symptoms, sometimes accompanied by fever or chills due to bacterial cholangitis [4]. For early-stage PSC, ursodeoxycholic acid is used to improve cholestatic parameters, whereas liver transplantation remains the definitive treatment for advanced disease.

#### Imaging findings

In PSC, diffuse chronic inflammation and fibrosis involving both intrahepatic and extrahepatic bile ducts lead to bile duct wall thickening and luminal narrowing. These changes subsequently result in segmental cholestasis and biliary dilatation.

**Fig. 6:**
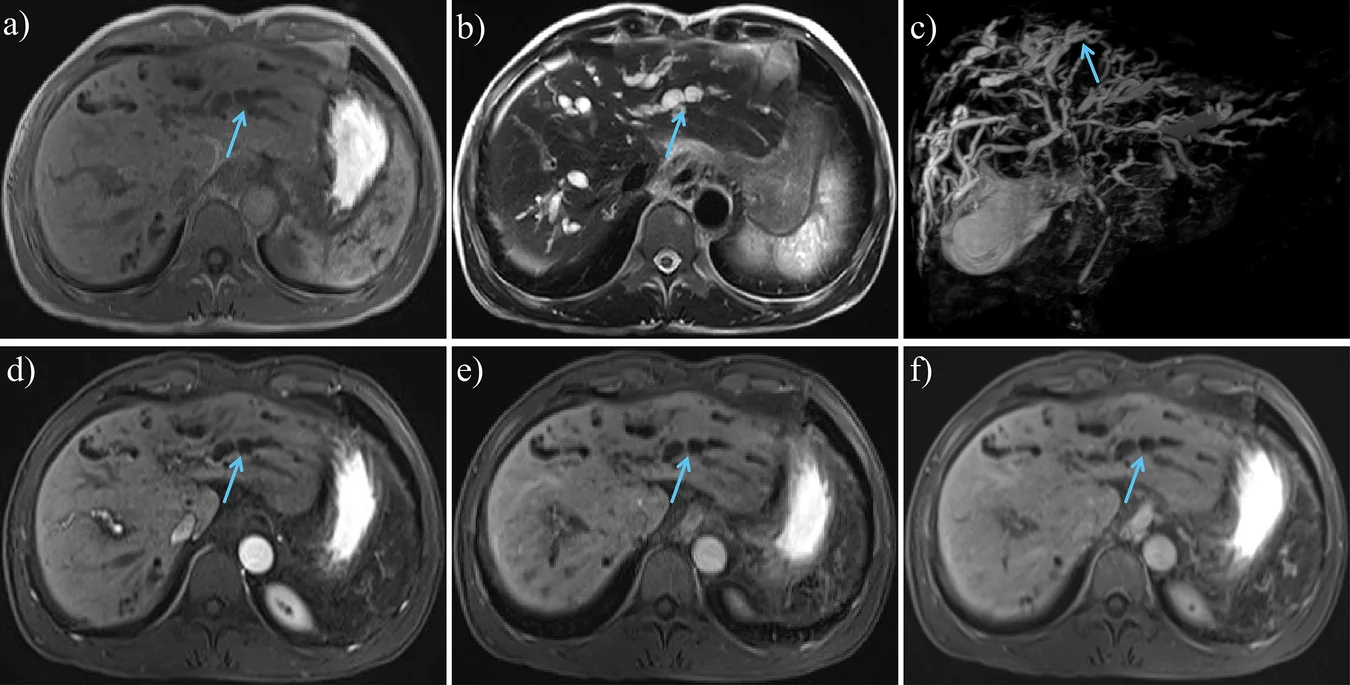
Imaging findings in a 54-year-old man with PSC. T1-weighted imaging (**a**), T2-weighted imaging (**b**), MRCP (**c**), arterial phase (**d**), portal venous phase, (**e**), and delayed phase (**f**) with gadoxetic acid disodium show multifocal and segmental dilation of the intrahepatic bile ducts, which presents a characteristic “beaded” and “pruned tree” appearance (arrows)

CT is not routinely used for the diagnosis of PSC. Instead, MRI or MRCP is preferred and typically demonstrates multifocal and segmental strictures of both intrahepatic and extrahepatic bile ducts, along with alternating bile duct dilatation, leading to a characteristic “beaded” appearance [4, 17] (Fig. 6). In some cases, PSC may present a “pruned tree” appearance due to the complete obstruction of small intrahepatic bile ducts [17, 34] (Fig. 6). When active inflammation is present, homogeneous thickening and enhancement of the bile duct wall may be identified on contrast-enhanced MRI, particularly during the arterial and portal venous phases. Saccular outpouchings, a distinctive imaging feature of PSC, are associated with a poor prognosis [34]. MR elastography can assess hepatic parenchymal stiffness in patients with PSC, providing valuable insights into disease progression and enabling the prediction of clinical outcomes [35]. Additional hepatic parenchymal changes associated with PSC include capsular retraction, segmental atrophy, and periportal fibrosis [34]. Diagnosis of PSC also requires close correlation with laboratory findings, particularly alkaline phosphatase and gamma-glutamyl transferase levels. In patients with PSC, several imaging features are highly suggestive of cholangiocarcinoma. These include focal periductal wall thickening accompanied by delayed enhancement, progressively enhancing perihilar masses, and vascular narrowing, all of which warrant further evaluation.

### IPNB

#### Definition and etiology

IPNB, first described by Chen in 2001 [36], is a relatively rare epithelial tumor characterized histopathologically by intraductal papillary epithelial proliferation and a delicate fibrovascular stalk [37]. IPNB is categorized into type 1 and type 2 based on its histological resemblance to intraductal papillary mucinous neoplasm (IPMN) of the pancreas. Type 1 IPNB closely resembles IPMN, whereas type 2 IPNB exhibits a more intricate histological pattern, featuring irregular papillary branching and areas of solid tubular components [38]. IPNB predominantly affects middle-aged and elderly men, with a higher prevalence in Asian countries, including China, Korea, and Japan [39, 40]. Although the exact pathogenesis of IPNB is not fully understood, it is generally considered to be strongly associated with underlying diseases such as hepatolithiasis, clonorchiasis, and PSC [37, 41]. These conditions can lead to recurrent biliary infections and cholestasis, potentially inducing abnormal proliferation of biliary epithelial cells, a key pathogenic mechanism in IPNB.

#### Clinical symptoms

Between 5% and 29% of patients with IPNB may remain asymptomatic [42]. As the tumor grows and mucin secretion increases, typical symptoms of IPNB, such as jaundice, abdominal pain, and cholangitis, may develop [40, 43]. Currently, surgical resection remains the preferred treatment for IPNB.

#### Imaging findings

**Fig. 7:**
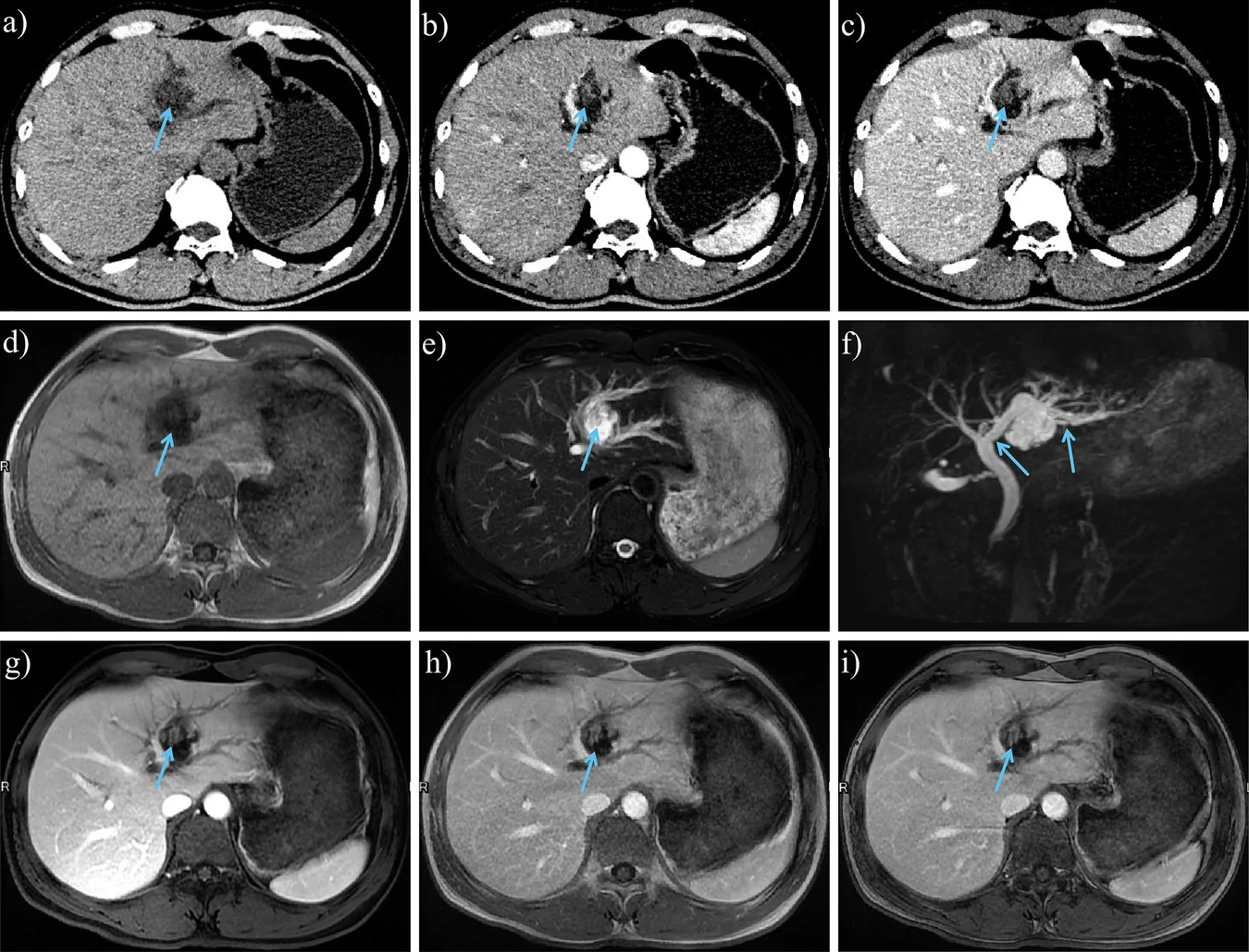
Imaging findings in a 49-year-old man with IPNB. Unenhanced CT (**a**) depicts a hypodense nodule within the intrahepatic bile duct. On arterial phase CT (**b**), the nodule demonstrates marked enhancement, whereas portal venous phase CT (**c**) shows a reduction in enhancement (arrow). T1-weighted imaging (**d**) reveals hypointensity of the nodule, and T2-weighted imaging (**e**) shows hyperintensity relative to the hepatic parenchyma (arrow). MRCP (**f**) demonstrates asymmetric dilation of the intrahepatic bile duct (arrow). Arterial phase (**g**), portal venous phase (**h**), and delayed phase MRI (**i**) with gadoxetic acid disodium show enhancement patterns consistent with the findings on CT (arrow)

On unenhanced CT, IPNB typically presents as intraductal masses accompanied by biliary dilatation. In the absence of solid components, the neoplasm manifests solely as intrahepatic biliary dilatation. Its solid components are isodense relative to hepatic parenchyma, whereas mucinous components show hypodensity similar to bile. In the late arterial phase, the lesion demonstrates heterogeneous enhancement, depending on mucin content and the proportion of solid tissues, followed by contrast washout during the portal venous phase. This enhancement pattern facilitates the differentiation of IPNB from other pathological entities, including cholangiocarcinoma [37, 44] (Fig. 7).

The solid component of IPNB shows hypointensity on T1-weighted imaging and isointensity/hyperintensity on T2-weighted imaging (Fig. 7). MRCP may show mucinous components as either indistinguishable from bile or as string-like filling defects, constituting the “thread sign” [17]. The degree of enhancement of the solid component varies, and such enhancement is generally more distinctly appreciated on MRI compared to CT [17]. Given that IPNB is a precursor of cholangiocarcinoma, tissue with solid components and restricted diffusion on diffusion-weighted imaging (DWI) should raise suspicion for malignant transformation. Studies suggest that factors such as the presence of an intraductal visible mass, tumor size ≥ 2.5 cm, multiplicity, bile duct wall thickening, and adjacent organ invasion are useful in diagnosing IPNB with associated invasive carcinoma [44].

Bile duct dilatation is a common feature of IPNB, resulting from either solid intraluminal papillary lesions or thick, viscous mucin. Type 1 IPNB often causes extensive dilatation involving both proximal and distal bile ducts due to mucus accumulation, whereas Type 2 IPNB leads to isolated proximal duct dilatation secondary to biliary obstruction [45] (Fig. 7). MRCP is highly effective in visualizing the extent of bile duct dilatation and elucidating the relationship between the tumor and the biliary tree.

MRI, particularly when using DWI and MRCP, is superior to CT for the detection and characterization of IPNB, as it provides superior soft tissue contrast resolution, evaluates tissue diffusion characteristics, and assesses morphological features of biliary duct dilatation. Although ultrasound can detect biliary dilatation and intraductal masses suggestive of IPNB, MRI/MRCP is superior for comprehensive assessment of lesion characterization, disease extent, and staging.

### ICC

#### Definition and etiology

ICC is a highly malignant tumor that originates from the epithelial cells of the bile ducts and is the second most prevalent primary liver cancer [46]. It can develop at any site along the biliary tree, from the canals of Hering to the main bile duct [47]. ICC typically arises in the setting of chronic biliary tract diseases [48]. Established risk factors for this malignancy include congenital biliary tract diseases [16], PSC [29], liver fluke infections [3], hepatolithiasis [23], and exposure to carcinogens [46]. Furthermore, at least four well-established genetic conditions—namely, BRCA-associated protein 1 (BAP-1) tumor predisposition syndrome, Lynch syndrome, cystic fibrosis, and biliary papillomatosis—have been identified as contributors to ICC susceptibility [46].

#### Clinical symptoms

Early-stage ICC is often asymptomatic. As the disease progresses, patients may develop symptoms such as jaundice, abdominal pain, weight loss, nausea, vomiting, and fever, along with elevated levels of the carbohydrate antigen 19-9 (CA19-9) [49].

#### Imaging findings

ICC can be categorized into four subtypes according to different growth patterns: mass-forming, periductal infiltrating, intraductal-growing, and mixed types [50]. Typically, mass-forming ICC exerts pressure on adjacent bile ducts, causing distortion and stenosis that ultimately lead to LIBD. Periductal infiltrating ICC grows longitudinally along the bile duct wall in an infiltrative manner, resulting in mural thickening and luminal stenosis. Intraductal-growing ICC is characterized by papillary or nodular lesions that induce bile duct stenosis or obstruction. Mixed-type ICC may involve overlapping mechanisms, including compression, infiltrative extension, and ductal obstruction, thereby causing pronounced LIBD.

**Fig. 8:**
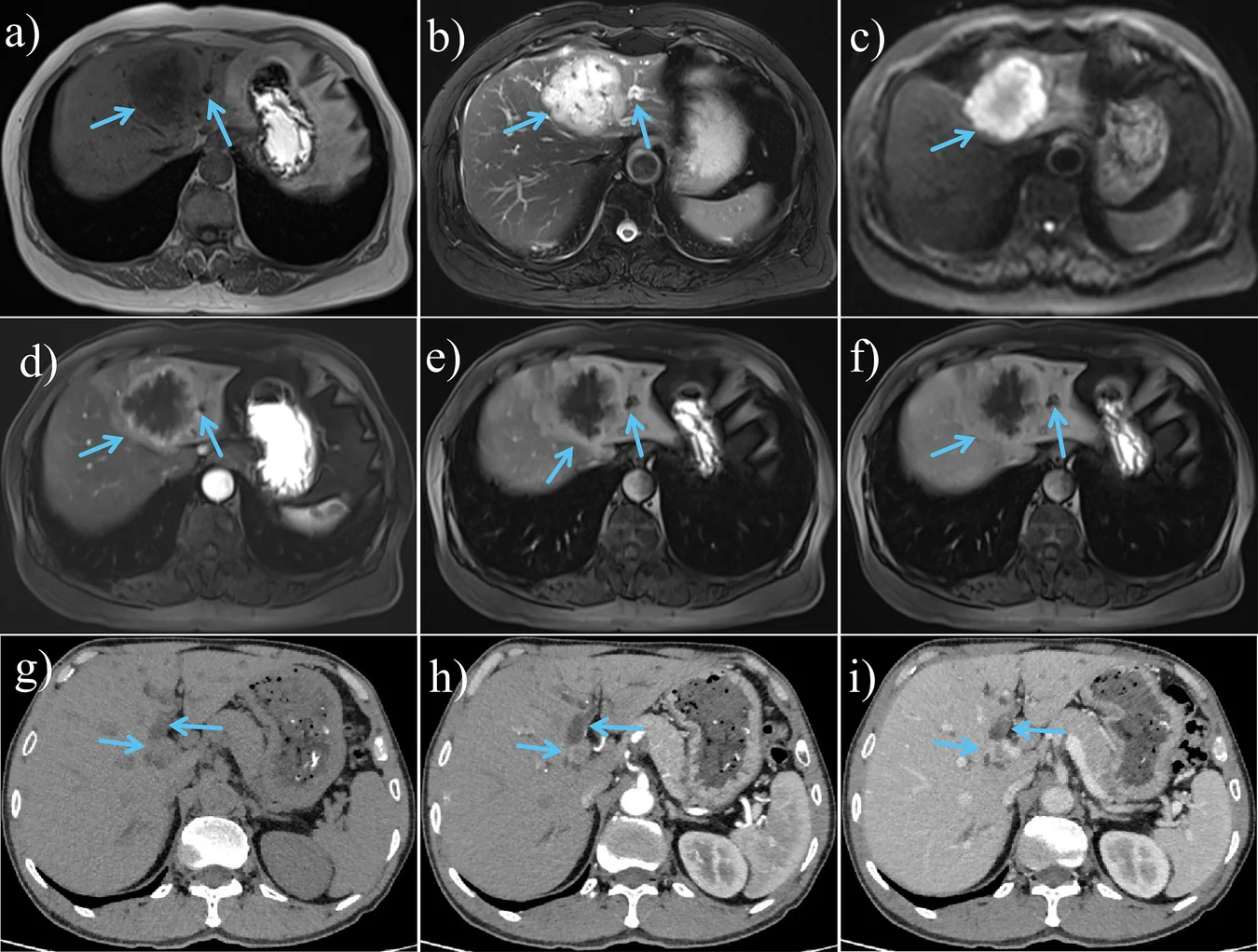
Imaging findings of ICC on CT and MRI. a–f show images of a 68-year-old female with mass-forming ICC. T1-weighted imaging (**a**) reveals a hypointense mass in segment IV of the liver, T2-weighted imaging (**b**) shows hyperintensity, and diffusion-weighted imaging (**c**) demonstrates a characteristic target sign. Gadoxetic acid disodium-enhanced MRI in the arterial phase (**d**) shows ring hyperenhancement, whereas portal venous phase (**e**) and delayed phase (**f**) images show progressive centripetal enhancement. LIBD is identified in the left hepatic lobe (arrow). **g**–**i** Show images of a 66-year-old man with periductal infiltrating ICC. Unenhanced CT (**g**) reveals intrahepatic bile duct stenosis with upstream biliary dilation. Arterial (**h**) and portal venous phase (**i**) CT demonstrate bile duct wall thickening with progressive enhancement, without evidence of a discrete mass (arrows)

Mass-forming ICC is typically characterized by irregular morphology, ill-defined borders, and non-encapsulated intrahepatic masses with dilated peripheral bile ducts. Its hallmark imaging features include an arterial phase ring hyperenhancement, followed by delayed progressive centripetal enhancement [51] (Fig. 8). This enhancement pattern is attributed to the presence of a fibrous stroma in the central portion of the tumor, which enhances slowly. Mass-forming ICC appears hypointense on T1-weighted imaging and demonstrates heterogeneous hyperintensity on T2-weighted imaging, depending on the extent of fibrosis, mucin content, and necrosis within the lesion [52]. Due to the presence of central fibrous stroma, DWI demonstrates a characteristic “target sign”, with mild diffusion restriction at the center of the tumor and marked restriction at the periphery [52–54] (Fig. 8). Smaller mass-forming ICCs may exhibit marked enhancement resembling HCC in the arterial phase on MRI or CT, but they do not demonstrate the “washout” enhancement in the portal venous phase, a finding that represents the characteristic imaging hallmark of HCC.

Periductal infiltrating ICC exhibits irregular thickening and progressive enhancement of the bile duct wall on CT or MRI [55] (Fig. 8). This subtype is often associated with significant proximal bile duct dilation, which may appear as a “soft vine-like” pattern. The bile duct wall thickening is typically eccentric and irregular, distinguishing it from benign conditions such as PSC and clonorchiasis. It is worth noting that the imaging features of periductal infiltrating ICC closely resemble those of biliary inflammatory pseudotumor, a rare benign inflammatory mass-forming lesion [56]. When imaging findings are indeterminate, correlation with clinical and laboratory data, short-term imaging follow-up, and/or image-guided biopsy is recommended to distinguish malignancy from biliary inflammatory pseudotumor.

Intraductal-growing ICC typically originates from IPNB, presenting as a polypoid intraluminal mass within the biliary tree. Although micropapillary lesions are often radiographically occult, they may be accompanied by segmental or lobar biliary dilatation. The mixed type, which combines mass-forming and periductal growth patterns, represents the second most prevalent morphological subtype of ICC. Although initially classified as a distinct entity, this subtype is now grouped under the mass-forming type due to the dominance of mass-forming features in its imaging phenotype [57].

CT is useful for assessing the extent of the tumor, vascular invasion, and distant metastases, while MRI is superior for detecting small lesions and differentiating ICC from other hepatic tumors. Accordingly, MRI constitutes the first-line imaging modality for small ICC and for cases that pose diagnostic challenges.

### Hepatocellular carcinoma (HCC) with bile duct tumor thrombus (BDTT)

#### Definition and etiology

HCC with BDTT was first reported by Mallory in 1947 [58] and later termed “Icteric-Type Hepatocarcinoma” in 1975 due to the presence of jaundice [59]. It is considered a distinct pathological subtype of HCC, accounting for approximately 0.53%–12% of all HCC cases [60]. The pathogenesis of BDTT remains a subject of debate and is believed to involve two primary pathways: direct formation of thrombus by cancer cells and cancer-associated thrombus resulting from blood coagulation triggered by invasive hemorrhage of the bile duct wall. The high invasiveness of primary HCC lesions, which are typically diffuse, poorly to moderately differentiated, and either lack a capsule or have only a partial capsule, is a key factor contributing to BDTT development [8, 61]. Studies have demonstrated that HCC with BDTT is associated with a poorer prognosis compared with conventional HCC, making accurate diagnosis crucial for the selection of appropriate treatment strategies [61]. Furthermore, it must be differentiated from other causes of biliary obstruction to prevent inappropriate management.

#### Clinical symptoms

Common clinical manifestations of HCC with BDTT include Jaundice, abdominal pain, liver dysfunction, and weight loss, with obstructive jaundice being the most frequent initial symptom. Laboratory tests often reveal significantly increased levels of alpha-fetoprotein (AFP), often exceeding 400 μg/L [60, 62].

#### Imaging findings

**Fig. 9:**
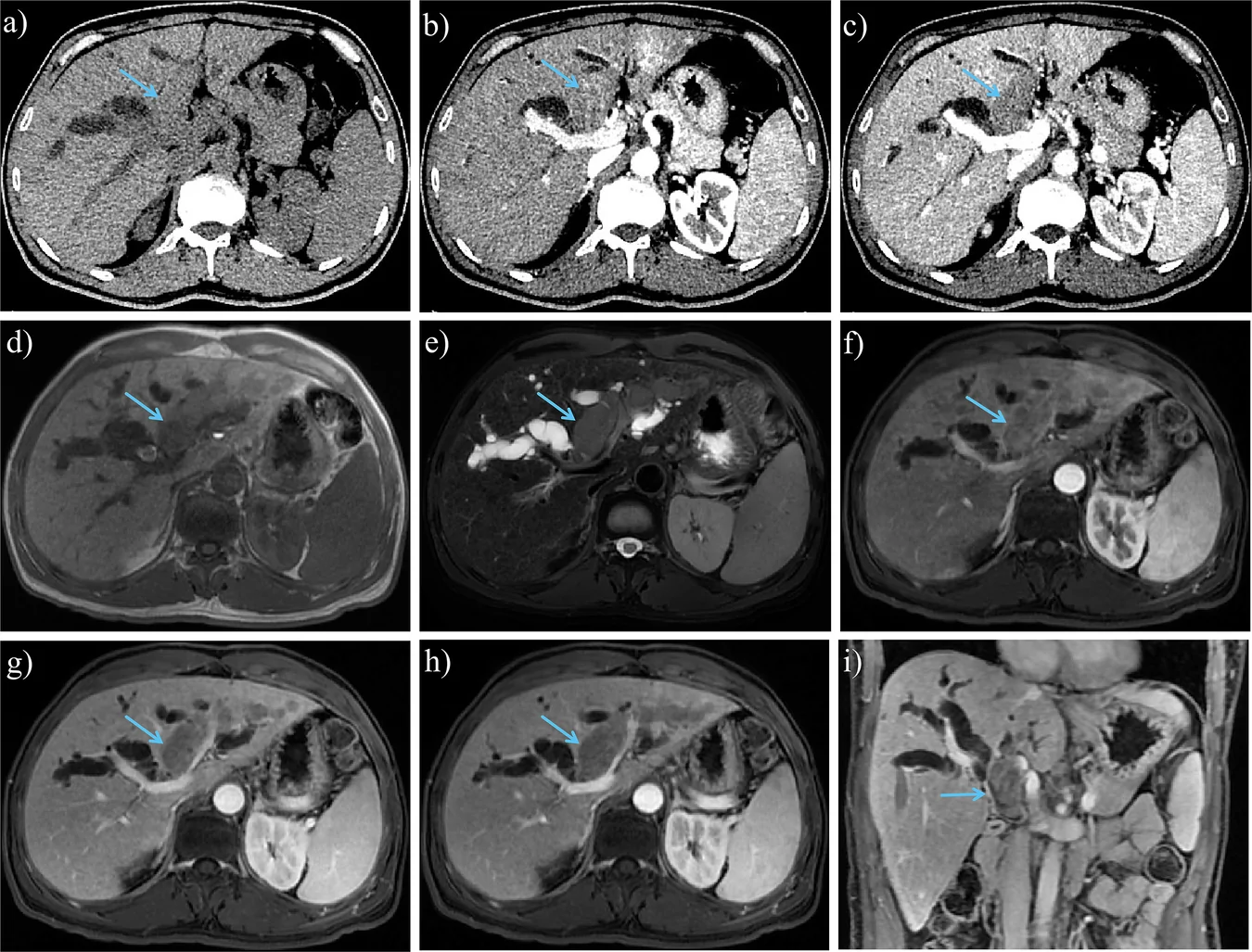
Imaging findings in a 51-year-old man with HCC complicated by BDTT. BDTT appears hypodense on unenhanced CT (**a**), exhibits marked enhancement on arterial phase CT (**b**), and demonstrates a “wash-out” appearance on portal venous phase CT (**c**, arrow). On T1-weighted imaging (**d**), the lesion shows hypointensity, whereas hyperintensity is noted on T2-weighted imaging (**e**) (arrow). Arterial phase (**f**), portal venous phase MRI (**g**), and delayed phase MRI (**h**) with gadoxetic acid disodium demonstrate enhancement patterns consistent with those observed on contrast-enhanced CT (arrow). Coronal portal venous phase MRI (**i**) reveals that the wall of the involved bile duct is smooth and continuous, without evidence of thickening or abnormal enhancement (arrow)

HCCs with BDTT consistently present with both parenchymal and intraductal lesions. Notably, the primary tumor can potentially induce thrombus formation even when the parenchymal lesion is small and undetectable on preoperative imaging [62, 63]. BDTTs typically appear as a hypodense or isodense mass within the bile duct on unenhanced CT. On contrast-enhanced CT, BDTT demonstrates a characteristic “wash-out” enhancement pattern, similar to the primary HCC lesion [62] (Fig. 9).

BDTT shows heterogeneous hyperintensity on T2-weighted imaging and restricted diffusion on DWI compared to the adjacent liver parenchyma [64]. On contrast-enhanced MRI, the lesion demonstrates the same enhancement pattern as observed on contrast-enhanced CT, distinguishing it from other biliary tract tumors (Fig. 9).

Another key feature of BDTT on CT or MRI is the appearance of the affected bile duct wall, which remains smooth, continuous, and free of thickening or abnormal enhancement [62] (Fig. 9). This feature helps differentiate BDTT from cholangiocarcinoma, which is typically characterized by irregular bile duct wall thickening, rigidity, and progressive enhancement. Due to direct occupation of the bile duct lumen, HCC with BDTT often causes severe LIBD. Studies have shown that intrahepatic bile duct dilation, as a potential diagnostic biomarker for BDTT in HCC patients, demonstrates a sensitivity of 93.33% and a specificity of 98.57%, respectively [8].

MRI outperforms CT in verifying BDTT presence, delineating its extent, and differentiating BDTT from alternative aetiologies of biliary obstruction. In addition, gadoxetic acid-enhanced MRI enables the identification of small primary HCC lesions that may evade detection on CT. Thus, MRI with MRCP constitutes the first-line imaging modality for the evaluation of HCC with BDTT.

### Other important causes of LIBD

In addition to the more common etiologies, LIBD may also arise from several clinically important secondary causes. Specifically, intraductal metastases most commonly originate from colorectal adenocarcinoma, where the intraluminal components cause proximal biliary obstruction and may also invade the biliary epithelium. Although differentiation between intrabiliary metastasis and ductal cholangiocarcinoma remains challenging, an expansile periductal mass is more likely in intrabiliary metastasis, whereas a periductal infiltrative pattern is more characteristic of ductal cholangiocarcinoma [65]. Extrinsic biliary strictures may occur secondary to compression by hilar lymphadenopathy, tumors of adjacent organs, or other intrahepatic mass lesions, and typically appear on imaging as luminal narrowing with upstream biliary dilatation. Contrast-enhanced MRI is particularly useful for demonstrating an intact bile duct wall and identifying surrounding compressive structures [66].

Ischemic cholangiopathy is another important cause of ILBD. It is characterized by biliary ischemia and necrosis due to inadequate hepatic arterial perfusion or hypoperfusion, with common causes including hepatic artery complications after liver transplantation, prior biliary operations, and transarterial interventional procedures. Imaging typically shows single or multiple short-segment non-anastomotic biliary strictures accompanied by ductal dilatation, and bilomas may also be present [17]. Portal biliopathy is seen in portal vein obstruction with cavernous transformation, where periportal collateral venous plexuses compress the bile ducts, leading to biliary tortuosity, focal strictures, and upstream dilatation. Its key imaging findings include abnormalities of the portal venous system and serpiginous periductal collateral vessels, which help distinguish it from cholangiocarcinoma [67]. Postoperative benign biliary strictures are also a common cause of LIBD following hepatic or biliary surgery. These strictures are typically confined to the anastomotic site or adjacent postoperative regions and usually present as smooth, gradually tapering narrowing without the irregular wall thickening characteristic of malignant infiltration [17]. The British Society of Gastroenterology guidelines emphasize that, when assessing biliary strictures or obstruction, benign causes—including ischemic cholangiopathy and portal hypertensive biliopathy—should be systematically considered and excluded to avoid misdiagnosis as cholangiocarcinoma [68].

## Structured differential diagnostic approach in common LIBD-associated diseases

**Fig. 10:**
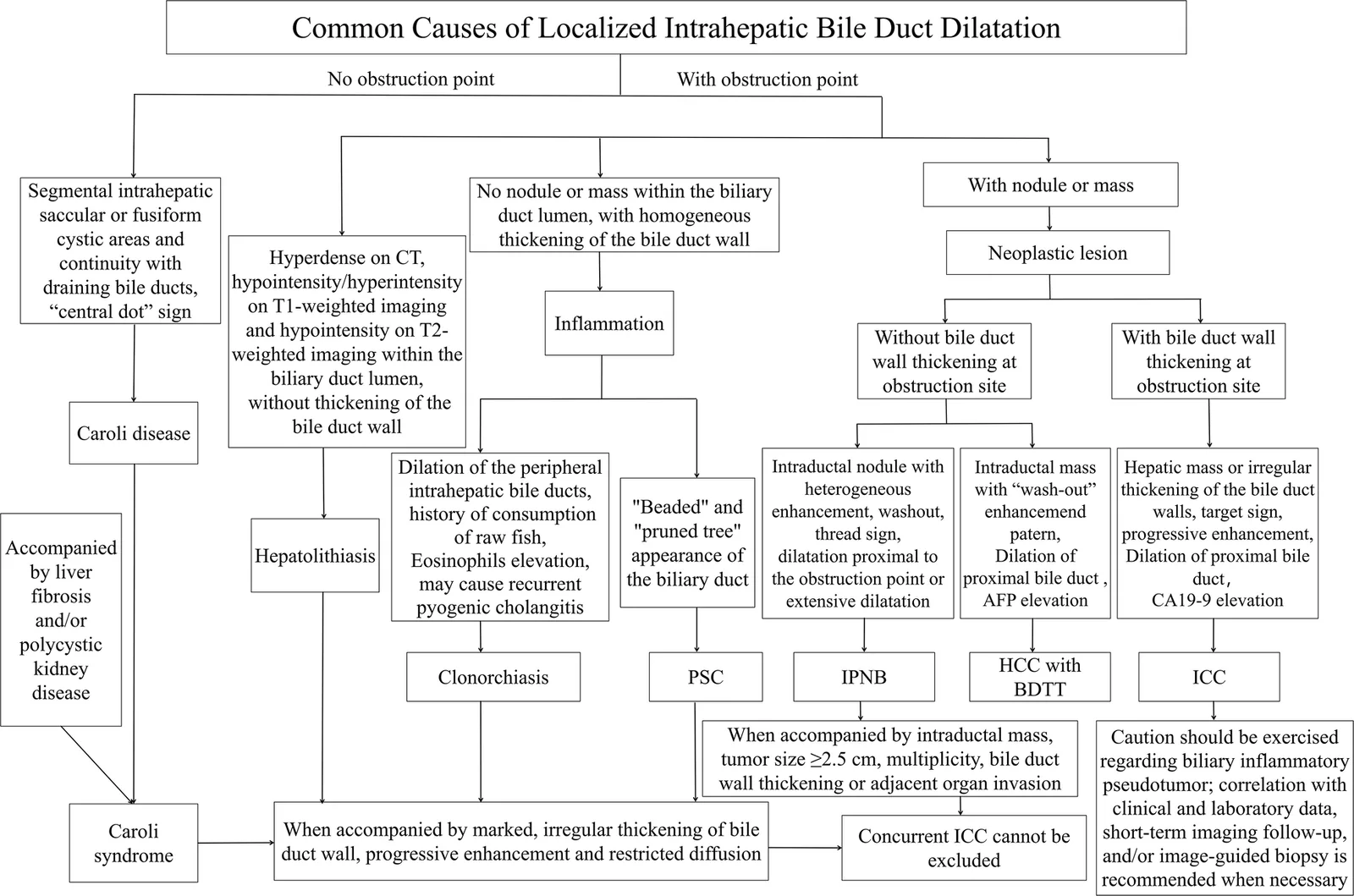
A structured differential diagnosis approach for common LIBD-associated diseases. CT, computed tomography; PSC, primary sclerosing cholangitis; IPNB, intraductal papillary neoplasm of the bile duct; HCC, hepatocellular carcinoma; BDTT, bile duct tumor thrombus; ICC, intrahepatic cholangiocarcinoma; AP, arterial phase; PVP, portal venous phase; DP, delayed phase; AFP, alpha-fetoprotein; CA19-9, carbohydrate antigen 19-9

A structured, stepwise approach is essential for navigating the diagnostic complexity of LIBD, given its heterogeneous etiologies and overlapping imaging phenotypes. This section outlines a practical framework based on two core pillars: the identification of “obstruction points” on cross-sectional imaging and the systematic integration of imaging findings with clinical features (Fig. 10). The goal is to guide radiologists toward accurate etiological differentiation, in alignment with the imaging correlates and clinical insights detailed in the preceding sections.

When no identifiable obstruction point is detected, communication between intrahepatic cystic lesions and the normal biliary tree, combined with the presence of a “central dot” sign, should raise suspicion of CD or CS. In contrast, for LIBD-associated diseases with a definite obstruction point, integrating additional imaging findings and clinical features is essential to improve the diagnostic accuracy for hepatolithiasis, inflammation, and neoplastic lesions (as shown in Fig. 10 and Table 1).

**Table 1:** Core features comparison of common LIBD-associated diseases

Diseases	CD	CS	Hepatolithiasis	Clonorchiasis	PSC	IPNB	ICC	HCC with BDTT
Obstruction point	No	No	Yes	Yes	Yes	Yes	Yes	Yes
Imaging features	Communication between intrahepatic cystic lesions and the normal biliary tree; central dot sign	Same biliary findings as CD; additional features including hepatic fibrosis, portal hypertension, and polycystic kidney disease	Hyperattenuation on CT, hypointensity/hyperintensity on T1-weighted imaging and hypointensity on T2-weighted imaging	Dilatation of peripheral intrahepatic bile ducts; homogeneous thickening and enhancement of bile duct walls; small intraductal filling defects	“Beaded” or “pruned tree” dilation of both intrahepatic and extrahepatic bile ducts; liver capsular retraction, segmental atrophy, and periportal fibrosis	Intraductal nodule with heterogeneous enhancement in AP and “washout” during PVP, thread sign	Hepatic mass or irregular thickening of the bile duct walls; progressive enhancement; target sign in mass-forming ICC	Primary HCC within the hepatic parenchyma, intraductal mass with “wash-out” enhancement pattern
Features of biliary duct dilatation	Cystic dilatation	Cystic dilatation	Proximal bile duct dilatation	Peripheral intrahepatic bile ducts dilatation	“Beaded” or “pruned tree” appearance of the dilated bile ducts	Type 1: dilation both proximal and distal to the obstruction point; Type 2: Proximal bile duct dilatation	Proximal bile duct dilatation	Proximal bile duct dilatation
Clinical symptoms/medical history	Fever, abdominal pain, jaundice, pruritus	Same symptoms as CD; Concurrent ARPKD, portal hypertension-related symptoms, renal insufficiency	Charcot’s triad or Reynold’s pentad in some cases; persistent jaundice and refractory ascites in advanced stages	History of consuming raw fish/shrimp; mild gastrointestinal disturbances; may cause recurrent pyogenic cholangitis	Young males predominate; concurrent inflammatory bowel disease (IBD); fluctuating jaundice, pruritus, and recurrent cholangitis	Jaundice, cholangitis; 5%–29% asymptomatic; associated with hepatolithiasis/clonorchiasis/PSC	Chronic biliary disease history; jaundice, weight loss	Obstructive jaundice as initial symptom; history of HCC
Laboratory examination	Normal	Normal	Normal	Eosinophils positive	ALP and GGT positive	Normal	CA19-9 positive	AFP positive

*CD* Caroli disease, *CS* Caroli syndrome, *PSC* primary sclerosing cholangitis, *IPNB* intraductal papillary neoplasm of the bile duct, *ICC* intrahepatic cholangiocarcinoma, *HCC* hepatocellular carcinoma, *BDTT* bile duct tumor thrombus, *CT* computed tomography, *AP* arterial phase, *PVP* portal venous phase, *ALP* alkaline phosphatase, *GGT* gamma-glutamyl transferase, *CA19-9* carbohydrate antigen 19-9, *AFP* alpha-fetoprotein

Notably, hepatolithiasis, clonorchiasis, PSC, and IPNB are all established risk factors for ICC. When imaging shows a coexistence of irregular bile duct wall thickening, progressive enhancement, and restricted diffusion in these conditions, ICC should be suspected. Looking ahead, advances in artificial intelligence, particularly technologies such as vision foundation models, are expected to provide substantial utility for the early detection of malignant transformation in these diseases.

## Conclusion

LIBD stems from a diverse array of etiologies, each associated with distinct treatment strategies and prognostic implications. Adopting a structured differential diagnostic approach that integrates imaging findings, clinical features, and laboratory data allows radiologists to make accurate diagnoses of LIBD-associated conditions and guides clinicians in implementing optimal clinical management.

## Data Availability

The datasets generated and analyzed during the current study are not publicly available because they contain sensitive patient information, but are available from the corresponding author upon reasonable request.

## Abbreviations

ARPKD: Autosomal recessive polycystic kidney disease
BDTT: Bile duct tumor thrombus
CD: Caroli disease
CS: Caroli syndrome
CT: Computed tomography
DWI: Diffusion-weighted imaging
HCC: Hepatocellular carcinoma
ICC: Intrahepatic cholangiocarcinoma
IPMN: Intraductal papillary mucinous neoplasm
IPNB: Intraductal papillary neoplasm of the bile duct
LIBD: Localized intrahepatic bile duct dilatation
MRCP: Magnetic resonance cholangiopancreatography
MRI: Magnetic resonance imaging
PSC: Primary sclerosing cholangitis
